# HLA-DRB1 and HLA-DQA1 associated with immunogenicity to adalimumab therapy in patients with rheumatoid arthritis

**DOI:** 10.1136/ard-2023-223955

**Published:** 2023-09-12

**Authors:** Chuan Fu Yap, Nisha Nair, Annick de Vries, Floris C Loeff, Ann W Morgan, John D Isaacs, Anthony G Wilson, Kimme L Hyrich, Anne Barton, Darren Plant

**Affiliations:** 1 Centre for Genetics and Genomics Versus Arthritis, Centre for Musculoskeletal Research, The University of Manchester, Manchester, UK; 2 NIHR Biomedical Research Centre, Manchester University NHS Foundation Trust, Manchester Academic Health Science Centre, Manchester, UK; 3 Diagnostic Services, Sanquin, Amsterdam, The Netherlands; 4 School of Medicine, University of Leeds, Leeds, UK; 5 NIHR Leeds Biomedical Research Centre, Leeds Teaching Hospitals NHS Trust, Leeds, UK; 6 NIHR In Vitro Diagnostic Co-operative, Leeds Teaching Hospitals NHS Trust, Leeds, UK; 7 Translational and Clinical Research Institute, Newcastle University, Newcastle upon Tyne, UK; 8 Musculoskeletal Unit, Newcastle-upon-Tyne Hospitals NHS Foundation Trust, Newcastle-upon-Tyne, UK; 9 School of Medicine and Medical Science, Conway Institute, University College Dublin, University College Dublin, Dublin, Ireland; 10 Centre for Epidemiology Versus Arthritis, Centre for Musculoskeletal Research, The University of Manchester, Manchester, UK

**Keywords:** Adalimumab, Autoantibodies, Methotrexate, Arthritis, Rheumatoid

Advanced targeted therapies including tumour necrosis factor inhibitors (TNFis) have transformed the clinical management of rheumatoid arthritis (RA). However, monoclonal antibody (MAb)-derived TNFis are associated with development of immunogenicity resulting in low circulating drug levels (online supplemental figure S5).^1^ A genetic predictor of immunogenicity would have clinical utility by providing a pretreatment biomarker that could be used to inform therapy selection. Previous genetic studies of TNFi immunogenicity have focused on alleles within the HLA locus on chromosome 6.^2–4^

10.1136/ard-2023-223955.supp1 Supplementary data

Patients were followed for 12 months with serum samples collected at 3 months, 6 months and 12 months following commencement on adalimumab (TNFi) therapy. Neutralising antidrug antibodies (ADAs) were detected using a drug-sensitive/drug-tolerant radioimmunoassay (Sanquin, NL). The presence of ADAs was determined by radioimmunoassay. A positive ADA titre was defined as >12 arbitrary units/mL. If a patient developed ADA at any time in the study, they were classed as ADA positive. Genotyping was carried out using the Illumina array, and HLA alleles were imputed using SNP2HLA and the T1DGC reference panel following standard data quality control (full details in online supplemental S1). Drug immunogenicity rates were determined using Kaplan-Meier analysis, and Cox proportional hazards regression, which was used to adjust genetic models for biological sex, age, concurrent conventional synthetic disease-modifying antirheumatic drug (csDMARD) use, disease duration and first within-sample principal component from the genetic dataset.

In total 445 patients were studied, of whom 96 (21.6%) became ADA positive during treatment. A total of 377 (85.3%) patients received cotherapy with csDMARDs of which 302 (81.4%) patients received methotrexate (MTX, online supplemental table S1). Disease duration modestly increased the rate of immunogenicity for every year since RA diagnosis (HR=1.02, p=0.01, table 1). Compared with TNFi monotherapy, combination therapy with csDMARD reduced the rate of ADA development by more than twofold (HR=0.379, p=1.27e−07). Importantly, a statistically significant difference in the rate of immunogenicity was observed when MTX cotherapy was compared with cotherapy with alternative csDMARDs; MTX conferring higher protection from immunogenicity (HR=0.425, p=1.27e−05). However, non-MTX csDMARD use also trended towards a reduced rate of immunogenicity (HR=0.66; 95% CI 0.429 to 1.012, p=0.056).

**Table 1:** Cox regression output for the clinical attributes, where N is the number of samples available within each variable

	N	P value	HR	ADA negative	ADA positive
Concurrent csDMARD usage	442	1.27e−07	0.38 (0.26–0.54)	354 (80%)	88 (20%)
Methotrexate (MTX) usage*	371	1.93e−05	0.41 (0.28–0.62)	312 (84%)	59 (16%)
MTX versus other csDMARD†	377	1.27e−05	0.43 (0.29–0.62)	315 (84%)	62 (16%)
Concurrent csDMARD (excluding MTX)	143	0.06	0.66 (0.43–1.01)	95 (66%)	48 (34%)
First biologic	444	0.88	0.95 (0.53–1.73)	356 (80%)	88 (20%)
Age	445	0.18	0.99 (0.97–1.00)	357 (80%)	88 (20%)
Sex	445	0.29	1.21 (0.85–1.73)	357 (80%)	88 (20%)
BMI	364	0.87	1.00 (0.97–1.03)	296 (81%)	68 (19%)
ACPA status	239	0.83	1.06 (0.65–1.72)	192 (80%)	47 (20%)
Never versus current smoker	151	0.27	0.66 (0.32–1.37)	125 (83%)	26 (17%)
Never versus ever smoker‡	254	0.47	0.85 (0.54–1.33)	207 (82%)	47 (18%)
Disease duration	438	0.01	1.02 (1.00–1.04)	351 (80%)	87 (20%)
Baseline DAS28 score	439	0.59	0.95 (0.78–1.15)	353 (80%)	86 (20%)

*Comparison within patients with complete MTX information, those with missing information were not included in this analysis.

†Comparison within recorded patients of having known combination therapy, as well as complete MTX information.

‡Ever smoker refers to ex smokers and current smokers.

ACPA, anti-citrullinated peptide antibody; ADA, antidrug-antibody; BMI, body mass index; csDMARD, conventional synthetic disease-modifying antirheumatic drug ; DAS28, disease activity score in 28-joints.

**Figure 1:**
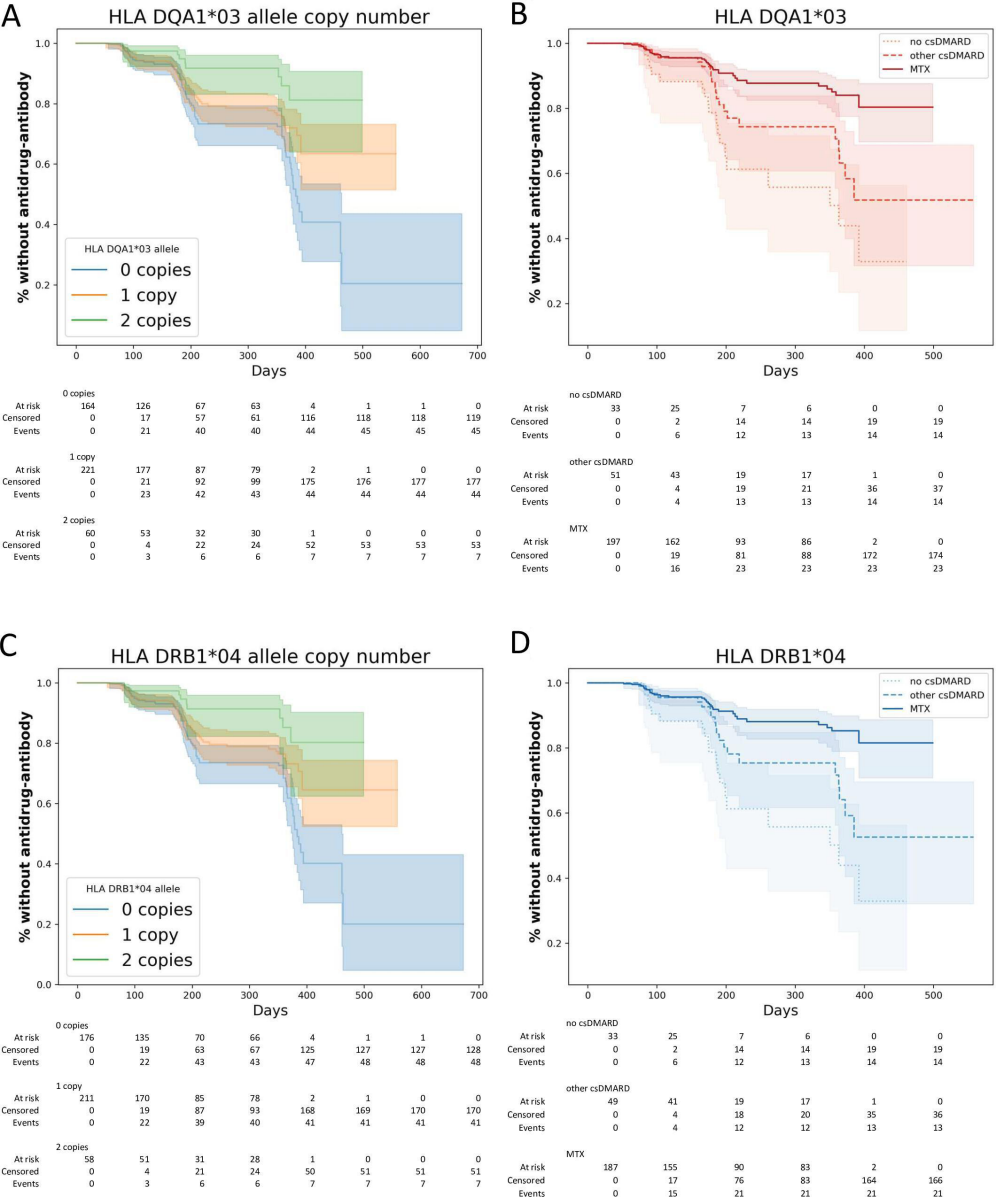
(A, C) Kaplan-Meier (KM) plot showing rate of drug antidrug antibody development, stratified by the number of HLA alleles carried (A, HLA-DQA1*03; C, HLA-DRB1*04). The tables presented underneath the KM plots represents the number of participants at risk over time. Blue, orange and green indicate 0, 1 and 2 copies of the alleles respectively. (B, D) Kaplan-Meier plot of drug immunogenicity rate for carriers of at least one copy of HLA-DQA1*03 and HLA-DRB1*04, respectively, for different types of csDMARD cotherapy. Solid line and darkest shade of colour represent cotherapy with MTX, dashed line and middle shade represents non-MTX csDMARD, dotted line with the lightest shade represents monotherapy with only adalimumab. csDMARD, conventional synthetic disease modifying antirheumatic drug; MTX, methotrexate.

Following quality control of the genetic data, 166 HLA alleles were available for analysis in 435 patients with non-missing covariate data. The most statistically significant association with immunogenicity was observed for HLA-DQA1*03 (HR 0.6; 95% CI 0.474 to 0.775, p=6.4e−05) and HLA-DRB1*04 (HR 0.6; 95% CI 0.476 to 0.775, p=6.3e−05) (4-digit and amino-acid results are reported in online supplemental material S1). In the Kaplan-Meier analysis, carriage of HLA-DQA1*03 and HLA-DRB1*04 alleles under an additive model was associated with reduced rate of immunogenicity (figure 1A–C). The two HLA alleles were in LD (R^2^: 0.94),^5^ suggesting a single protective effect. In carriers of at least one copy of HLA-DQA1*03 or HLA-DRB1*04, MTX was observed to provide stronger protection against ADA development compared with other csDMARDs (HR 0.44; 95% CI 0.24 to 0.78, p=5.7e−03, figure 1B–D). We also investigated HLA alleles that have previously been reported on in RA and Crohn’s disease and provide support for alleles at HLA-DQA1*05, HLA-DRB1*11 and HLA-DRB1*03 (online supplemental figure S4).

In conclusion, in the largest study of its type in RA to date, carriage of HLA-DQA1*03 and HLA-DRB1*04 reduced the rate of drug immunogenicity to adalimumab. The strongest protection from immunogenicity was conferred by csDMARD cotherapy, particularly in combination with MTX. Our results suggest that the use of alternative csDMARDs should be encouraged for patients treated with MAb TNFi who are MTX intolerant. Larger studies are now needed to determine if genetic testing could optimise selection of treatment and to quantify effects of non-MTX csDMARDs on immunogenicity.
