# Supplementary material for: HLA-DRB1 and HLA-DQA1 associated with immunogenicity to adalimumab therapy in patients with rheumatoid arthritis
Source: Ann Rheum Dis. 2023 Sep 12;83(2):263–5. doi: 10.1136/ard-2023-223955 (PMC10850714; doi:10.1136/ard-2023-223955)
Supplement: Supplementary data [file ard-2023-223955supp001.pdf]

Supplementary Document

Table of contents

1. Methods Patients
2. Methods, Genotype sample processing details
3. Methods, Cox regression model.
4. Table 1, Patient characteristics
5. Table 2, Statistical output for final Cox regression model for HLA-DRB1, DQA1 and DQB1
6. Amino acid Manhattan plots.
7. Forest Plot of HLA alleles reported in other literature
8. Non-trough Drug levels
9. References

Patients

The patients in this study were already taking part in the Biologics in Rheumatoid Arthritis Genetics and Genomics Study Syndicate (BRAGGSS, Research Ethics Committee (REC) reference: 04/Q1403/37), which is a prospective multi-centre observation study cohort based in the UK. BRAGGSS patients included in the current study had a diagnosis of RA according to the American College of Rheumatology 1987 revised criteria for the classification of RA [1], were of European ancestry and were about to receive treatment with adalimumab for their RA symptoms. Adalimumab was the anti-TNF agents most commonly prescribed for the treatment of RA in the national UK cohort at the time that this study was designed. In total 671 adalimumab treated patients were recruited from 2008-2019 where a genetic sample was available for genotyping, 1 patient was withdrawn from the study due to non-compliance. Of these 671's patients, 445 of had a serum sample at the 6-month follow-up visit of sufficient quality and quantity to permit testing for anti-drug antibodies. The category of non-MTX csDMARDs included: leftlunomide (n=6), sulphasalazine (n=8), Azathioprine (n=4) and hydryxocyhloroquinne (n=1). These csDMARDs were grouped together in the analysis due to missing data and, where the csDMARD was known, low numbers of individuals receiving the different drugs.

Genotype sample processing

Genotyping was carried out using the Illumina Infinium HumanCoreExome 24 BeadChip kit (Illumina, San Diego, California, USA). 250 ng of DNA was used, according to the manufacturer's guidance. Genotype calling was carried out using GenomeStudio software (Illumina, San Diego, California, USA). Standard QC was conducted on each individual array using PLINK v1.9 [2]: SNPs and samples were excluded if there was >2% missing data, and SNPs with MAF < 0.01 and Hardy Weinberg Equilibrium (HWE) $p < 1 \times 10^{-4}$ were also excluded. Population stratification adjustment was done using HapMap 3 reference panel [3], that includes individuals of European descent, to determine genetic ancestry of each individual, followed by Principal Component Analysis (PCA) analysis. Only individuals of European descent were kept in the dataset. HLA information (types and amino acid) were imputed using SNP2HLA using T1DGC reference panel; imputation refers process of assigning SNP that were not genotyped in the array using a reference panel, the SNPs would then be assigned amino acids, and subsequently allele types [4].

Cox Regression Model

Cox proportional hazards regression model was used to determine immunogenicity rate association to HLA alleles using an additive genetic model. The final genetic model was adjusted for biological sex, age, concurrent conventional synthetic disease modifying anti-rheumatic drug (csDMARD) use, disease duration, and first within-sample principal component from the genetic dataset. After accounting for all available data that includes the above covariates, there were only 435 samples left. Smoking information which could be informative was excluded as there was high number of missing relative to the entire cohort (43%); inclusion of this variable would greatly reduce the power of the study. Another potentially informative variable to be excluded was BMI, this was also due to high number of missing data (18%). However, a final model that included BMI was built, the inclusion of

BMI information did not alter the results for either HLA-DRB1 or HLA-DQA1 in comparison to the same subset of patients where BMI was excluded from the model. For statistical testing the p-value threshold for significance was set to 3E-04. This value is derived from dividing p=0.05 by the number of alleles tested (n=166).

			Grouped by Antidrug-Antibody Status		
		Missing	Overall	Negative	Positive
n			445	349 (78.4)	96 (21.6)
Sex, n (%)	Female	0	337 (75.7)	263 (75.4)	74 (77.1)
	Male		108 (24.3)	86 (24.6)	22 (22.9)
Age, mean (SD)		0	57.2 (11.9)	57.5 (11.7)	56.0 (12.9)
Disease Duration, median [Q1,Q3]		7	6.6 [2.5,15.4]	5.9 [2.5,14.1]	9.3 [3.0,17.9]
First Biologic, n (%)	No	1	66 (14.9)	57 (16.4)	9 (9.4)
	Yes		378 (85.1)	291 (83.6)	87 (90.6)
Baseline DAS, mean (SD)		6	5.2 (0.9)	5.2 (0.9)	5.2 (0.9)
Concurrent csDMARD usage, n (%)	No	3	65 (14.7)	36 (10.4)	29 (30.2)
	Yes		377 (85.3)	310 (89.6)	67 (69.8)
MTX treatment, n (%)	No	74	69 (18.6)	47 (15.3)	22 (34.9)
	Yes		302 (81.4)	261 (84.7)	41 (65.1)
Smoking Status, n (%)	Current smoker	191	43 (16.9)	37 (18.3)	6 (11.5)
	Ex-smoker		103 (40.6)	82 (40.6)	21 (40.4)
	Never smoked		108 (42.5)	83 (41.1)	25 (48.1)
BMI, median [Q1,Q3]		81	27.8 [24.2,32.5]	28.1 [24.6,32.6]	26.1 [23.6,31.2]
ACPA+ve, n (%)	No	206	62 (25.9)	50 (26.3)	12 (24.5)
	Yes		177 (74.1)	140 (73.7)	37 (75.5)
Time Points, n (%)	3 months	0	144 (32.4)	114 (32.7)	30 (31.2)
	6 months		107 (24.0)	76 (21.8)	31 (32.3)
	12 months		194 (43.6)	159 (45.6)	35 (36.5)

Table S1: Patient characteristics summary for this study. *tableone* package was used to generate patient characteristics table [5].

VARIANT	GENE	*p*-value	HR	Lower CI	Higher CI	Patients with 1 Copy	Patients with 2 Copies
HLA-DRB1*04	DRB1	0.000063	0.607	0.476	0.775	204	57
HLA-DRB1*0404	DRB1	0.000241	0.327	0.18	0.594	31	2
HLA-DRB1*0401	DRB1	0.004429	0.626	0.453	0.864	136	3
HLA-DQA1*0301[+]	DQA1	0.000064	0.606	0.474	0.775	214	59
HLA-DQA1*03[+]	DQA1	0.000064	0.606	0.474	0.775	214	59
HLA-DQB1*02	DQB1	0.000326	1.622	1.246	2.111	115	18

HLA-DQB1*03	DQB1	0.001195	0.685	0.545	0.861	207	94
HLA-DQB1*0302	DQB1	0.005412	0.637	0.463	0.875	152	14
HLA-DQB1*0202	DQB1	0.006217	1.692	1.161	2.467	57	0
HLA-DQB1*0201	DQB1	0.025259	1.468	1.049	2.054	73	6

Table S2: Statistical output for final Cox regression model for HLA-DRB1, DQA1. Given that DQA1 and DQB1 form a heterodimer, the results for tested alleles at the DQB1 locus are also presented. However, the results for DQB1*02 or DQB1*03 did not meet the p-values the threshold for multiple testing (p=3E-04). ⁺The results for HLA-DQA1*03 and HLA-DQA*0301 were identical because there is only one 4-digit allele for HLA-DQA1*03.

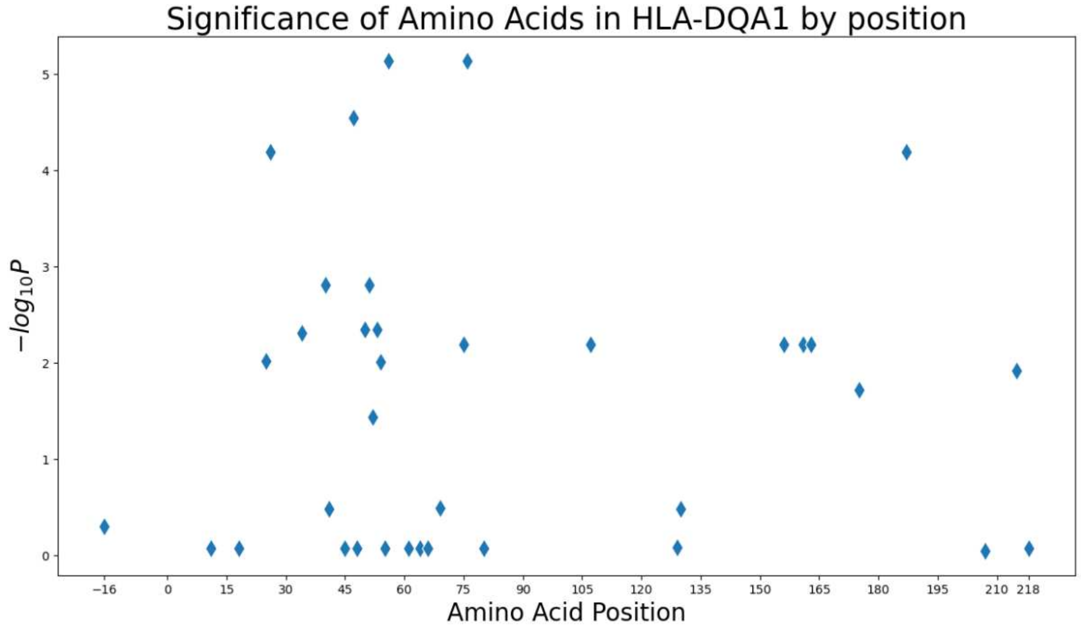

Figure S1: Manhattan plot for HLA-DQA1 amino acids. Most significant positions are 56 and 76.

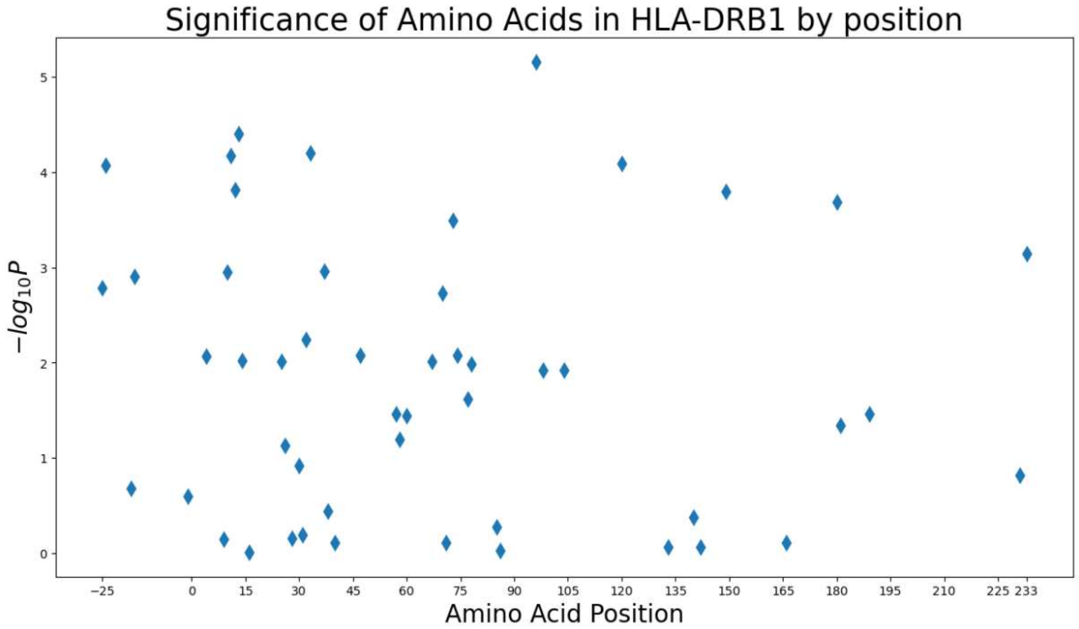

Figure S2: Manhattan plot for HLA-DRB1 amino acids. Most significant positions is 96.

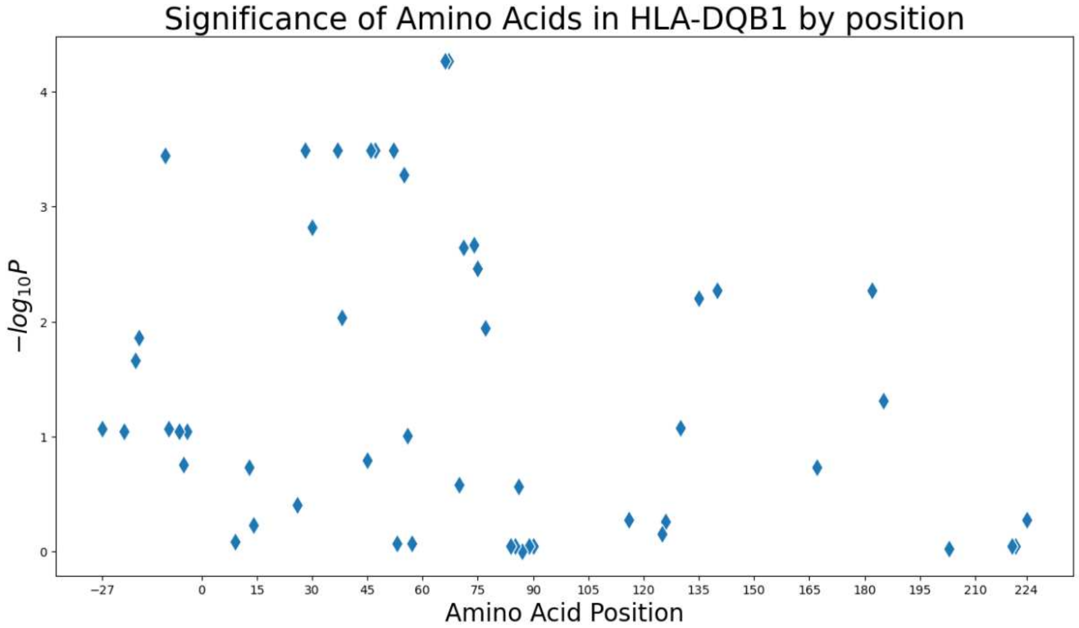

Figure S3: Manhattan plot for HLA-DQB1 amino acids. Most significant positions are 66 and 67.

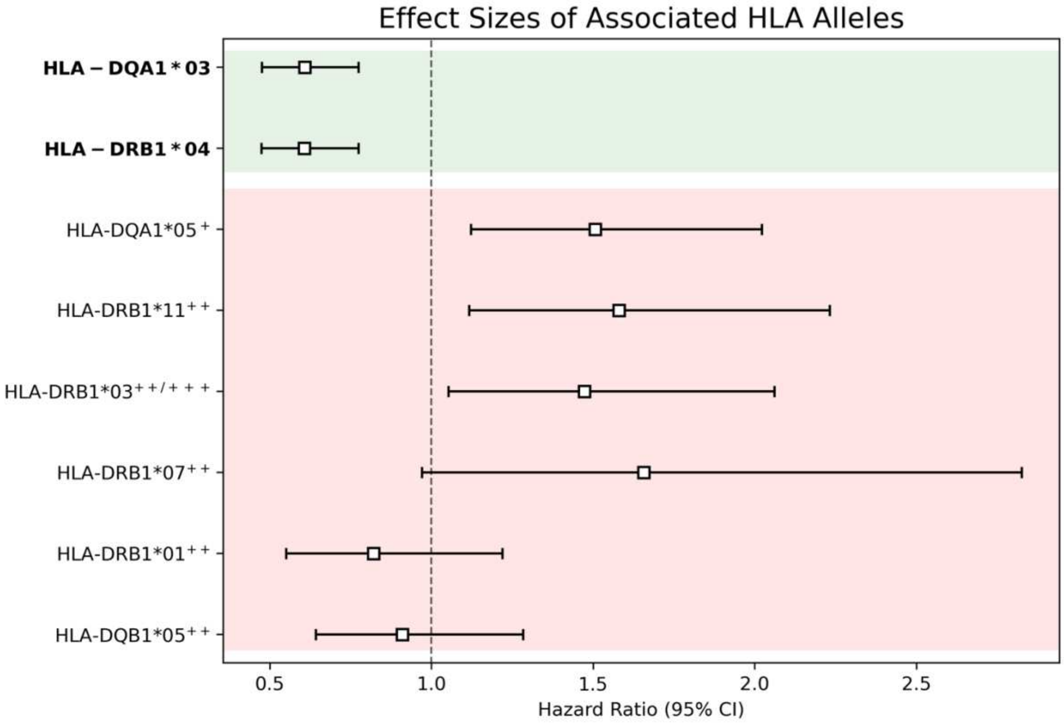

Figure S4: Effect sizes of HLA alleles on time to immunogenicity. The region marked in green are the alleles with the strongest association found in this study. The region marked in red are associations found in other studies but with effect sizes (and standard error) measured from the current study. For alleles found in other study, all alleles except for HLA-DRB1*07, were in the same direction, i.e. those found to be risk, were also found to be risk here. [+]From Sazonovs et al [6], [++]From Liu et al [7] and [+++]From Billiet et al [8].

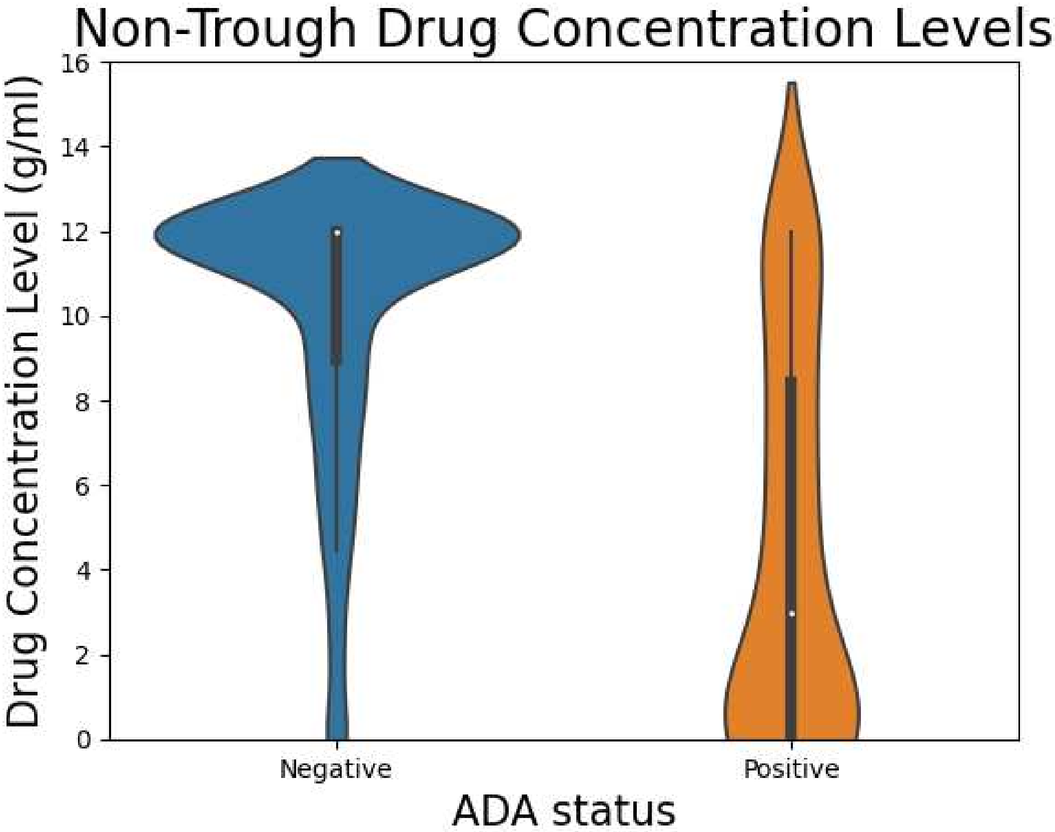

Figure S5: Violin plot for non-trough drug levels for ADA negative and positive samples. Mann-Whitney test indicated statistically significant difference with *p*: 5.7e-33.

References

1 Arnett FC, Edworthy SM, Bloch DA, *et al.* The american rheumatism association 1987 revised criteria for the classification of rheumatoid arthritis. *Arthritis Rheum* 1988;**31**:315–24. doi:10.1002/art.1780310302

2 Purcell S, Neale B, Todd-Brown K, *et al.* PLINK: A Tool Set for Whole-Genome Association and Population-Based Linkage Analyses. *Am J Hum Genet* 2007;**81**:559–75. doi:10.1086/519795

3 HapMap3. https://www.sanger.ac.uk/resources/downloads/human/hapmap3.html

4 Jia X, Han B, Onengut-Gumuscu S, *et al.* Imputing Amino Acid Polymorphisms in Human Leukocyte Antigens. *PLoS One* 2013;**8**:e64683. doi:10.1371/journal.pone.0064683

5 Pollard TJ, Johnson AEW, Raffa JD, *et al.* tableone: An open source Python package for producing summary statistics for research papers. *JAMIA Open* 2018;**1**:26–31. doi:10.1093/jamiaopen/ooy012

6 Sazonovs A, Kennedy NA, Moutsianas L, *et al.* HLA-DQA1*05 Carriage Associated With Development of Anti-Drug Antibodies to Infliximab and Adalimumab in Patients With Crohn's Disease. *Gastroenterology* 2020;**158**:189–99.

doi:10.1053/j.gastro.2019.09.041

7 Liu M, Degner J, Davis JW, *et al.* Identification of HLA-DRB1 association to adalimumab immunogenicity. *PLoS One* 2018;**13**:e0195325. doi:10.1371/journal.pone.0195325

8 Billiet T, Vande Casteele N, Van Stappen T, *et al.* Immunogenicity to infliximab is associated with HLA-DRB1. *Gut* 2015;**64**:1344–5. doi:10.1136/gutjnl-2015-309698
